# Restoration of Degraded Soil in the Nanmangalam Reserve Forest with Native Tree Species: Effect of Indigenous Plant Growth-Promoting Bacteria

**DOI:** 10.1155/2016/5465841

**Published:** 2016-04-18

**Authors:** Andimuthu Ramachandran, Parthasarathy Radhapriya

**Affiliations:** Centre for Climate Change and Adaptation Research, College of Engineering, Anna University, Guindy Campus, Chennai 600025, India

## Abstract

Restoration of a highly degraded forest, which had lost its natural capacity for regeneration, was attempted in the Nanmangalam Reserve Forest in Eastern Ghats of India. In field experiment, 12 native tree species were planted. The restoration included inoculation with a consortium of 5 native plant growth-promoting bacteria (PGPB), with the addition of small amounts of compost and a chemical fertilizer (NPK). The experimental fields were maintained for 1080 days. The growth and biomass varied depending on the plant species. All native plants responded well to the supplementation with the native PGPB. The plants such as* Pongamia pinnata, Tamarindus indica, Gmelina arborea, Wrightia tinctoria, Syzygium cumini, Albizia lebbeck, Terminalia bellirica,* and* Azadirachta indica* performed well in the native soil. This study demonstrated, by using native trees and PGPB, a possibility to restore the degraded forest.

## 1. Introduction

Forest degradation is an increasing worldwide problem that leads to poor soil health [1]. Natural regeneration and revegetation are very slow in a highly disturbed forest soil. Revegetation with native flora is one of the anticipated solutions to combat degradation [2]. One of the basic theories explaining the repeated failure of natural revegetation of eroded areas and the difficulties in establishing planted or seeded native plants is based on the premise that the top soil has lost its beneficial plant-associated microorganisms and, consequently, part of its fertility and growth potential [3, 4]. For the establishment of plants in eroded soil, water alone is not sufficient to recover the loss of soil fertility and microbial communities. At least some essential plant growth-promoting microorganisms should be artificially reintroduced [5, 6]. Plant growth-promoting bacteria (PGPB) are beneficial in harsh and limiting environments because of their role in improving stress tolerance in plants [7]. PGPB are considered to be a vital element of plant and soil interactions in eroded soil [6, 8–10]. PGPB inoculation along with fertilizer has been reported to increase dry matter accumulation and nutrient uptake in the* Fraxinus americana* forest seedlings [11]. Bashan et al. [6] emphasized that the best approach to find PGPB compatible with degraded soils is to isolate, proliferate, and use native PGPB from the soil or plants already growing there. PGPB improve the soil quality through improving plant and soil interactions and increasing plant biomass.

In tropical ecosystems, soil degradation is a key threat to sustainable soil use, because there is a great variation in the spatial and temporal accessibility of water and nutrients, and dominant plants alter soil properties that lead to intricating local interactions between soil and vegetation [12]. Usually, soil physicochemical properties are analyzed to assess soil quality [13]. However, these properties evolve slowly and, thus, considerable changes only occur over many years. By contrast, soil microbial and enzymatic properties react relatively quickly to small changes in the soil environment, and it has been broadly reported that any changes in soil management and land use are reflected in soil enzyme activities [14, 15]. Additionally, soil enzyme activities can reveal alterations in soil quality before the latter are detected by other means of soil analysis [16]. Therefore, microbial and enzyme properties are considered to be early indicators of future soil changes. Soil microbial metabolic processes cannot be measured based on individual properties, so different authors established a rhizosphere soil microbial index (RSMI) using principal component analysis (PCA) to assess the rhizosphere soil quality and suggested that tree species with higher RSMI values are most suitable for replanting degraded soils [17–19].

In India, most forests are in a degraded state due to various need-based forestry practices (e.g., felling of timber for construction, fuel, or the creation of grazing lands and real estate) used for over 150 years [4]. In Eastern Ghats of India, application in the past of various forest practices reflecting different mindsets of the foresters led to massive removal of tree species, leaving only remnants of what used to be a forest [4, 20]. The forest devastation went in parallel with soil degradation due to a continuous reduction in canopy and to soil erosion. Reduced litter formation and removal of top soil lead to deterioration of the site and unavailability of nutrients, soil organic carbon (SOC), and microbial load [21]. The Nanmangalam Reserve Forest (NRF) in Tamil Nadu state, India, is one of such forests, wherein top soil has completely vanished and few indigenous species have been in existence with stunted growth for years. It is hypothesized that native tree species and indigenous PGPB could effectively improve the rhizosphere soil. The present study aimed to restore the degraded forest using PGPB and indigenous tree species.

## 2. Study Area and Climatic Conditions

The NRF is located in the southern part of Chennai and spreads in an area of 321 ha (12°55′5′′N to 12°56′13′′N and 80°9′46′′E to 80°10′57′′E) southwest of Chennai, adjoining the coastal track of the Bay of Bengal. The study area was a fragmented hill of Eastern Ghats of Tamil Nadu. The average rainfall (2012–2014) in the forest was 161.1 mm, the maximum temperature was 30.4 to 32.3°C, and the minimum temperature was 25 to 26.1°C. The forest soil has clayey texture, with the following physicochemical characteristics: bulk density, 1.02 g/cm^3^; pH 6.8; organic carbon, 1.10%; nitrogen, 0.19%; available phosphorus, 1.66 mg kg^−1^; available potassium, 121.51 mg kg^−1^; calcium, 7.6 mg kg^−1^; magnesium, 3.92 mg kg^−1^; iron, 0.31 mg kg^−1^; manganese, 0.28 mg kg^−1^; copper, 0.28 mg kg^−1^; and zinc, 0.16 mg kg^−1^.

## 3. Materials and Methods

### 3.1. Microorganisms and Plants

In this study, a consortium of native plant growth-promoting bacteria was used. The consortium contained 10^8^ cells of* Burkholderia* sp. RRAK1,* Pseudomonas* sp. RRAN2,* Azospirillum* sp. RRAK5,* Paenibacillus* sp. RRB2, and* Bacillus* sp. RRN12. Partial sequences of the 16S rRNA gene of the organisms were submitted to the GenBank of the National Center for Biotechnology Information under accession numbers KJ137015, KF531968, KF531969, KF952292, and KF952292, respectively. The bacteria were selected and used in the NRF for replantation with the following 12 plant species selected:* Albizia lebbeck*,* Azadirachta indica*,* Gmelina arborea*,* Madhuca latifolia*,* Pongamia pinnata*,* Pterocarpus santalinus*,* Syzygium cumini*,* Tamarindus indica*,* Terminalia arjuna*,* Terminalia bellirica*,* Thespesia populnea*, and* Wrightia tinctoria*. The plants common name was given in S. Table 1 (see Supplementary Material available online at http://dx.doi.org/10.1155/2016/5465841). These are the predominant tree species growing in the NRF. To compare the effect of the tree type on soil microbial properties, the tree species without PGPB was used as a control. The soil strongly adhering to roots and within the space explored by the root system was considered to be rhizosphere soil. The soil samples were taken from the ground under three randomly selected trees of each species, and each sample was divided into two parts. One part was air dried for analysis of soil physicochemical properties, whereas the second was immediately sieved through a 2 mm mesh and then stored at 4°C until analysis of biological properties.

### 3.2. Inoculum Preparation and Application

The five native PGPB were cultivated in a nitrogen-free medium for 72 h at room temperature on a shaker at 120 rpm, and the cultures were mixed with a lignite carrier. All the bacterial inoculants mixed with lignite were used to treat seedlings of the forest trees at 10^8^ CFU g^−1^ of soil. Cow manure compost was mixed with soil in a proportion of 1 : 8 (compost : soil, 735 g : 10 kg, w/w) [6], and the mixture was added to each planting hole. A mixture of soil and the bacterial inoculants (1 kg) was used to inoculate each tree species. The inoculants and compost for the field experiment were mixed into the soil by hand. This mixture was stored in 25 kg sacks at ambient temperature until being used (not to exceed two weeks). In control soil compost was added, without mixing of PGPB.

### 3.3. Seed Collection, Screening of Seedlings Cultivated, and Planting

Seeds of the above tree species were collected from the Nanmangalam nursery, Chennai, Tamil Nadu, India. Seedlings for transplantation to field plots were cultivated in polyethylene bags for 11 months and were of a similar height for each species and the size of the polyethylene bags was 30 × 45 cm. The total plots size was 1 acre. Triplicates of ten plants for each species were planted. The planting pits were designed to maximize the contact between inoculants and plants and conserve water. Each transplant was planted in an identical pit, with a depth of 25 cm and a diameter of 30 cm. The soil extracted was mixed with the bacterial inoculants and compost and refilled to the pit. Each pit received 7 kg of this inoculated soil on the day of planting and after one year. The experiment was conducted for 24 months (March 2012 to March 2014). The above-ground biomass was estimated every six months based on the girth and shoot height. After 24 months, soil physicochemical and biological characteristics were analyzed (Table 1).

### 3.4. Rhizosphere Soil Microbial Index (RSMI)

The RSMI was determined as described by Masto et al. and Bastida et al. [30, 31]. The index method involved three key steps: (1) selection of suitable soil physicochemical and biological properties; (2) transformation and weighing of the properties; and (3) combining the scores into an index.

The choice of appropriate properties and their weighting was determined by PCA. In each principal component (PC), only the properties loading higher values were chosen for indexing; high-factor loadings were defined as having absolute values within 10% of the highest factor loading [32]. If there was more than one property with high loading in a single PC, only the properties that did not correlate with each other were considered to be important and were therefore selected. If the properties were well correlated, the one with higher loading was selected for the determination of the RSMI [17]. To transform microbial property values into scores, (1) defining a sigmoidal-type curve was used [30, 31]:

(1) $$\begin{array}{c} S=\frac{a}{\left(1+{\left(x/x_{0}\right)}^{b}\right)}, \end{array}$$

where *S* is the score of the proposed property after transformation; *a* is the maximum score (in this case, *a* = 1); *x* is the value of the microbial property; *x*_0_ is the mean value of each microbial property; and *b* represents the slope value of the equation.

In the PCA, there are two kinds of properties, one is greater than 0 (more is better) and the other is less than 0 (less is better), to obtain a sigmoidal curve tending to 1 for all proposed properties. If the eigenvalue is >0, then the value of *b* is 2.5, and if the eigenvalue is <0, the value of *b* is −2.5:

(2) $$\begin{array}{c} RSMI=\overset{n}{\underset{i=1}{\sum }}W_{i}S_{i}, \end{array}$$

where *W* is the weighting factor of microbial properties derived from the PCA. Since not all properties have the same importance in determining the rhizosphere soil quality, each PC explains a certain amount of variance (%) in the total dataset, which provides the weight for the properties chosen in a PC [17, 30]. The equation was finally normalized to get a maximum RSMI equal to 1.

### 3.5. Statistical Analysis

All the results were reported as the mean ± standard deviation. The differences between the mean values were evaluated using a one-way analysis of variance (ANOVA). Comparison among the means was performed using the Duncan multiple range test, calculated at *p* < 0.05 and *p* < 0.01. Correlation analysis, scoring, and PCA were conducted using the SPSS software version 20.0.

## 4. Results

**Figure 1:**
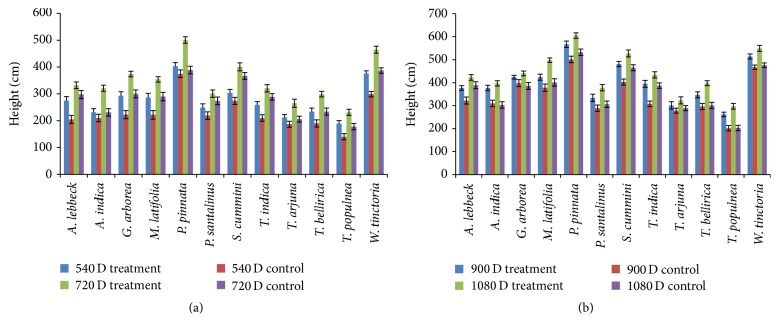
(a) Above-ground heights of the 12 different plant species in field experiment using application of the PGPB consortium and control for 540–720 days. (b) Above-ground heights of the 12 different plant species in field experiment using application of the PGPB consortium and control for 721 to 1080 days.

**Figure 2:**
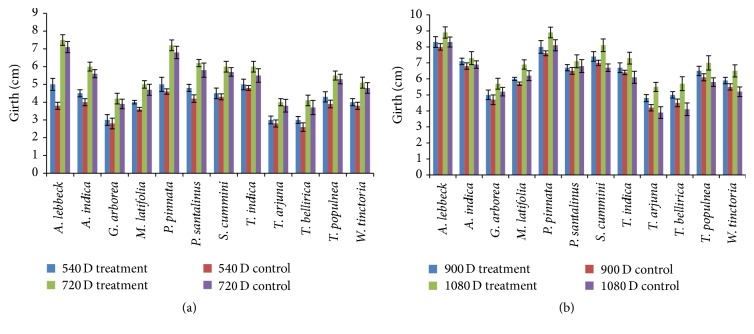
(a) Girth of the 12 different plant species in field experiment using application of the PGPB consortium and control for 540 days to 720 days. (b) Girth of the 12 different plant species in field experiment using application of the PGPB consortium and control for 721 days to 1080 days.

Growth of the selected native tree species inoculated with PGPB was measured at the same intervals (once in 6 months). Based on the height,* P. pinnata* occupied the first place, followed by* T. indica*,* G. arborea*, and* W. tinctoria*, and the least growth was demonstrated by* T. arjuna*,* T. bellirica*, and* T. populnea* (Figures 1(a) and 1(b)). Girths of all plant species were measured, and the largest girths were observed in* A. lebbeck*,* P. pinnata*, and* S. cumini*, whereas the smallest girths were observed in* G. arborea*,* T. arjuna*, and* T. bellirica* (Figures 2(a) and 2(b)). Based on the growth (height and girth) of the tree species, it was confirmed that their biomass increased in comparison to that of the control. These observations could in turn be a potential tool for enhancing growth of all the native tree species from the NRF that were used in this study.

### 4.1. Soil Chemical and Biological Properties

The pH values of the replanted rhizosphere soils ranged from 5.9 to 6.9. The lowest pH value was observed under* T. indica*, and the maximum pH value was observed under* P. pinnata*. There were no significant differences among the rhizosphere soils of the revegetation species. The EC values ranged from 0.1 to 0.37 for the rhizosphere soils of the different tree species. SOC slightly increased in the rhizosphere of the treated soils and ranged from 0.9 to 1.5%. The lowest SOC value was observed in the control plant. The maximum SOC value was observed under* P. pinnata*, followed by* W. tinctoria*. A similar pattern was observed for total nitrogen contents of all rhizosphere soils, which ranged from 0.048 to 0.059%. MBC tended to be highest in the rhizosphere soils of* T. indica* (543 mg kg^−1^) and* P. pinnata* (533 mg kg^−1^), followed by that of* S. cumini* (511 mg kg^−1^). The lowest value was recorded under* T. populnea* (303 mg kg^−1^). The MBN values varied from 56 mg kg^−1^ to 91 mg kg^−1^ in the* treated* plant soil (S. Figure 1). The microbial biomass (MBC/MBN) ratios varied from 4.01 to 7.20 under the different plant species, and there was no significant difference (*p* = 0.34) among the tree species (Table 2). The microbial quotients (MBC/SOC) varied between 25.78 and 39.31 and differed significantly (*p* < 0.001) among the revegetation plants. The metabolic quotients (BSR/MBC) ranged between 0.009 and 0.022, and the highest ratio was observed for* P. pinnata*, while the lowest ratios were observed for* T. populnea* and* P. santalinus*. BSR/MBC differed significantly (*p* < 0.05) among the vegetated soils. The basal soil respiration rates of the rhizosphere soils differed significantly among the different tree species, ranging from 8.9 mg CO_2_–C kg^−1^ day^−1^ for* T. arjuna* and 6.5 mg CO_2_–C kg^−1^ day^−1^ for* G. arborea* to 3.5 mg CO_2_–C kg^−1^ day^−1^ in the control (S. Figure 1).

### 4.2. Microbial Enzymes

The rhizosphere soils collected under the vegetation types differed significantly for all microbial enzymes tested. The highest values for urease were observed in the rhizosphere of* P. pinnata* and* T. indica*, followed by that of* G. arborea*. The soil phosphatase levels ranged in the rhizosphere soils from 95.4 to 284.12 mg PNP g^−1^ h^−1^. The highest phosphatase level was found in the rhizosphere of* P. pinnata* (284.12 mg PNP g^−1^ h^−1^). The *β*-glucosidase levels ranged from 27.8 mg PNP g^−1^ h^−1^ to 87.8 mg PNP g^−1^ h^−1^. The highest values were recorded for* T. bellirica*,* T. indica,* and* P. pinnata*. The dehydrogenase levels of the PGPB treated plants were 38 to 87 *μ*g INF g^−1^ 2 h^−1^. The levels of phenol oxidase ranged from 0.01 *μ*M g^−1^ h^−1^ in the rhizosphere soils of* A. indica* and* S. cumini* to 0.08 *μ*M g^−1^ h^−1^ under* P. pinnata*. The catalase activity ranged from 257 to 548 *μ*g of H_2_O_2_ consumed per g of soil per h and varied widely among the tree species, indicating the differential oxidative and oxidoreductive potentials of the rhizosphere (S. Figure 2).

### 4.3. Rhizosphere Soil Microbial Index

The rhizosphere soil properties with significant differences (*p* < 0.05) among the revegetated sites were selected using PCA. Four PCs had eigenvalues > 1. In PC1, the properties with higher loading were EC, MBC, urease, *β*-glucosidase, dehydrogenase, and BSR/MBC. Urease positively correlated with SOC (S. Table 2), but in PC1 urease had a higher loading factor, so SOC was excluded from the RSMI. Dehydrogenase and *β*-glucosidase also positively correlated with each other but the former had a higher loading factor and was added to the RSMI. The BSR/MBC ratio was also highly loaded in PC1 and added to the RSMI. BSR, MBC/MBN, and MBC/SOC from PC2 (Table 3) were also included in the RSMI. PC1 and PC2 when combined together accounted for 72% of variation. In this study, the RSMI was established to evaluate the rhizosphere soil quality in the NRF for the 12 tree species inoculated with the consortium of the five different PGPB. Seven microbial properties that were considered to be the most crucial indicators were ultimately selected for indexing through PCA to express the overall soil quality. They were EC, urease, dehydrogenase, the metabolic quotients of BSR/MBC, MBC/MBN, and MBC/SOC, and basal soil respiration. The scores and weights of the properties were examined by two different equations (1) and (2) given in Methods (Table 4). The polynomial for obtaining the RSMI was as follows:

(3) RSMI=0.471+x0.202.5+0.4711+x/53.99−2.50+0.4711+x/53.15−2.50+0.4711+x/0.0152.5+0.2111+x/4.91−2.5+0.2111+x/1.67−2.5+0.2111+x/34.66−2.5.

**Figure 3:**
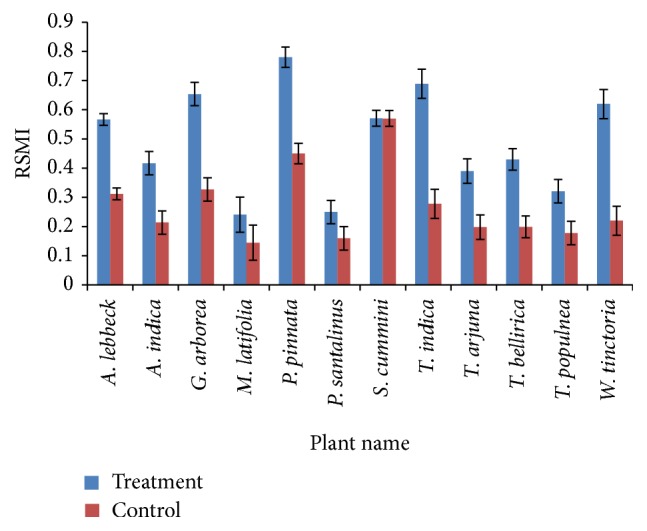
RSMI values for the different tree species in the NRF.

The RSMI values ranged from 0.25 to 0.78 in PGPB treated plants (Figure 3).

## 5. Discussion

Presently, the foremost risk to replantation in forests is topsoil loss due to erosion. This makes a situation in which there is a decrease in the number of beneficial plant-associated microorganisms present to sustain plant growth [33]. The above-average rainfall alone is not enough to promote good plant growth and to enhance the microbial population even in the case of desert soils [6]. Thus, the planned reintroduction of native PGPB is an ideal solution for plant and soil nutrient enhancement in degraded forests [34]. This study clearly explained the enhancement of plant biomass and soil when applied for native PGPB in degraded forest soils.

The acidic pH of the plant rhizosphere soils may be due to the release of acidic exudates by the revegetated tree species. This result is in agreement with the data obtained by Shen et al. and Chen et al. [35, 36]. The EC variation observed in the rhizosphere soils may be due to differences among plant root exudates. Generally, root exudates change the salinity of the rhizosphere soil [33]. Usually, addition of SOC to soil is vital and its loss may result in worsening of the soil structure [18]. SOC and TN as significant factors in the build-up and enhancement of soil MBC and MBN provide sufficient C, N, and energy sources to support microbial growth. In this study, higher values of SOC and TN in the plant rhizosphere correlated with increased MBC and MBN values. Microbial biomass varied considerably between the different plant species, suggesting that the MBC level was influenced by plant-microbe interactions. In rhizosphere, the colonization of microbes is affected by several factors, including the amount and quality of root exudates excreted by a particular plant species, physicochemical properties of the soil, and climatic conditions. In addition, some plant species secrete inhibitory substances that are toxic to soil microflora, which also affects the levels of MBC and MBN. There is no strong evidence that this is the only mechanism behind the relationship between tree species and soil microflora [37]. The microbial quotient (MBC/SOC) reflects the amount of the substrate available and the portion of total soil C immobilized in microbial cells. It is the most sensitive indicator of both microbial biomass and organic C levels [38]. In this study, no positive correlation was observed between the SOC and MBC/SOC values, which is in agreement with the results of Garcia et al. [38]. The MBC/MBN ratio is reflective of the structure and state of a microbial community. BSR is generally used to measure microbial activity [39]. A high BSR/MBC ratio indicates that the soil microbial activity is low and that the soil microorganisms are under environmental stress [18]. In the NRF revegetated rhizosphere soils, the BSR/MBC values were very low under* P. pinnata* and* T. indica*. The lower metabolic quotient in the rhizosphere soil indicated a good soil quality of the rhizosphere.

Enzymes are crucial soil biological components involved in the dynamics of a soil nutrient change. They are considered to be early indicative signs of changes in soil quality, because of their rapid response to the changes [40]. The soil enzymes measured in this study were urease, phosphatase, *β*-glucosidase, phenol oxidase, dehydrogenase, and catalase. Higher values were recorded for the enzymes in the rhizosphere soils compared to the control soil. Catalase and phenol oxidase varied widely among the tree species, indicating the differences in the respective oxidative and oxidoreductive potentials of the tree rhizosphere soils. This has been confirmed by Sinha et al. [17] for a coal mining ecosystem. The differences in the rhizosphere soil enzyme activities observed among the different tree species might be attributed to either different quantities of MBC or different microbial populations in the rhizosphere soils. Urease activity plays a crucial role in soil C and N cycling and has been used in different studies to assess the overall soil quality in various ecosystems [40, 41]. In this study, the soil urease levels were high compared to the control.

Several studies have established RSMIs to evaluate the rhizosphere soil quality under different revegetation plants [30, 31]. In this study, seven soil properties, which were considered to be the most decisive indicators for overall soil quality, were selected for indexing using PCA. Those were EC, urease, dehydrogenase, metabolic quotients of BSR/MBC, MBC/MBN, and MBC/SOC, and basal respiration. These properties were finally defined for RSMI indexing in our study. Other researchers have unveiled rhizosphere soil microbial indices in their studies [17–19]. In this study, different tree species were characterized by diverse RSMI values, which were in the range from 0.25 to 0.78 for the microbial consortium-treated plants, whereas the control plants showed a much lower RSMI value of 0.16–0.57 (Figure 3). Based on the data, the RSMIs could be segregated into three groups, with high (>0.600), medium (0.400–0.600), and low (<400) values. High RSMIs were observed for* P. pinnata*,* T. indica*,* G. arborea*, and* W. tinctoria*, medium RSMIs were observed for* S. cumini*,* A. lebbeck*,* T. bellirica*, and* A. indica*, and low RSMIs were observed for* T. arjuna*,* T. populnea*, and* P. santalinus*. Based on the RSMI values reported in another study [18],* A. indica*,* B. bauhinia,* and* Butea monosperma* are suitable trees for systematic plantations for coal mining land reclamation, and the use of RSMIs would reduce the risk of low success rates in tree establishment. Here, it is recommended that tree species having RSMI value of >0.5 could be used for tree establishment in the degraded forest of the NRF.

Lopez-Lozano et al. [42] clearly explained that beneficial interactions between PGPB and plants established by long term restoration are a promising venue for degraded soil reforestation, by not only long term survival of the trees but also their potential contribution to improve the N_2_ fixation in degraded soils by increasing the population of diazotrophs.

## 6. Conclusions

The above results clearly indicate that the consortium of PGPB, including* Burkholderia* sp. RRAK1,* Pseudomonas* sp. RRAN2,* Azospirillum* sp. RRAK5,* Paenibacillus* sp. RRB2,* and Bacillus *sp. RRN12 used in combination with six tree species,* P. pinnata*,* T. indica*,* G. arborea*,* W. tinctoria*,* S. cumini*, and* A. lebbeck* (RSMI > 0.5), can enhance the biomass in the restoration of the degraded NRF soil. The observation of the plant growth and the rhizosphere soil microbial index calculated clearly demonstrated that the application of PGPB and selected tree species supported better plant biomass and soil quality in the degraded forest in the NRF when compared to the control treatment.

## Supplementary Material

Supplementary Table 1: Plants' Scientific name and their common name in Nanmangalam Reserve Forest.
Supplementary Table 2: Correlation matrix between the different properties determined.
Supplementary Figure 1: pH, EC, SOC, TN, MBC/MBN, and soil respiration values obtained for rhizosphere soil samples of the 12 different tree species.
Supplementary Figure 2: Rhizosphere soil enzymes (urease, phasphatase, *β*-Glucosidase, dehydrogenase, phenoloxidase, Catalase) levels under the 12 different tree species.

## Figures and Tables

**Table 1:** Methods for soil characteristics.

Estimation	Method
Soil properties	
Bulk density (BD)	Core method [22]
Soil pH	Potentiometry 1 : 25 (soil-water) [22]
Electrical conductivity (EC)	Conductometry (1 : 2 soil-water suspension) [22]
Biological properties	
Soil organic carbon (SOC)	CHSN/O Elemental Analyzer [23]
Microbial biomass carbon (MBC)	Fumigation extraction method [24]
Microbial biomass nitrogen (MBN)	Fumigation extraction method [24]
Soil basal respiration	Estimation of CO_2_ evolved during the incubation of soil in a closed system [25]
Urease activity	Determination of ammonia released after the incubation of soil samples with urea solution for 2 hr at 37°C [26]
Phosphatase activity	Determination of p-nitrophenol released after the incubation of soil with p-nitrophenyl phosphate for 1 hr at 37°C [26]
*β*-glucosidase activity	The determination of the released p-nitrophenol after the incubation of soil with p-nitrophenyl glucosidase solution for 1 hr at 37°C [27]
Phenol oxidase	To measure phenol oxidase enzyme activity, 3,4-dihydroxy-L-phenylalanine (DOPA) was used as a substrate. The mixture was incubated at 20°C for 1 h. After that, it was centrifuged, and absorbance of the supernatant was measured at 460 nm using a UV spectrophotometer [18]
Dehydrogenase	Estimation of TTC reduction of soils [28]
Catalase	Determination of H_2_O_2_ consumed by the soil [29]

**Table 2:** Statistical significance analysis of the rhizosphere soil properties.

Serial number	Properties	*F*-significance	Standard error *n* = 3
1	pH	0.007	0.034
2	EC	0.005	0.02
3	Microbial biomass carbon	<0.001	11.1
4	Microbial biomass nitrogen	<0.001	2.2
5	Soil organic carbon	<0.001	0.5
6	Total nitrogen	<0.005	0.032
7	MBC/MBN	0.34	0.476
8	MBC/SOC	0.001	0.564
9	BSR/MBC	<0.001	0.009
10	Urease	0.005	2.21
11	Phosphatase	0.200	0.65
12	*β*-glucosidase	0.004	0.52
13	Phenol oxidase	<0.001	0.012
14	Dehydrogenase	0.000	3.49
15	Catalase	0.011	2.12

**Table 3:** PCA analysis of soil microbial parameters under the rhizosphere of different tree species.

Parameters	Components			
Variables	1	2	3	4
Variation (%)	46.631	24.755	9.567	8.029
Eigen value	6.427	3.313	2.527	1.330
Cumulative variations	46.631	67.386	76.95	84.98
pH	−0.564	−0.318	−0.257	0.043
EC	0.941	0.488	−0.575	−0.038
SOC	0.741	0.279	−0.039	0.132
TN	0.411	0.384	0.355	−0.065
MBC	0.501	0.103	0.069	−0.104
MBN	0.437	0.521	−0.214	−0.025
Soil basal respiration	−0.132	0.855	0.521	−0.143
Urease	0.902	−0.243	0.113	−0.490
Phosphatase	0.351	0.064	0.273	−0.234
*β*-glucosidase	0.614	−0.136	−0.263	0.017
Phenol oxidase	0.015	−0.088	0.453	0.345
Dehydrogenase	0.820	−0.388	−0.015	0.265
Catalase	0.421	0.297	−0.229	0.285
MBC/MBN	0.141	−0.947	0.137	0.052
MBC/SOC	0.348	−0.804	−0.356	0.252
BSR/MBC	−0.739	0.538	0.355	−0.037

EC: electrical conductivity; SOC: soil organic carbon; TN: total nitrogen, MBC: microbial biomass carbon; MBN: microbial biomass nitrogen.

Extraction method: principal component analysis.

Four components extracted.

RSMI = 0.47 EC + 0.47 urease + 0.47 dehydrogenase + 0.47BSR/MBC + 0.21BSR + 0.21MBC/MBN + 0.21MBC/SOC.

Normalized RSMI = RSMI/2.51.

Final RSMI = 0.176EC + 0.169 urease + 0.153 dehydrogenase + 0.138 BSR:MBC + 0.0717BSR + 0.079MBC:MBN + 0.067MBC:SOC.

**Table 4:** Average values, values range, and *R* ^2^ coefficients of scoring value for different parameters.

Properties	EC	Urease	Dehydrogenase	BSR:MBC	Basal respiration	MBC:MBN	MBC:SOC
Average (*x*°)	0.20	53.99	53.15	0.014731	4.91	1.67	34.66
Curve type	Less is better	More is better	More is better	Less is better	More is better	More is better	More is better
Slope (*b*)	2.5	−2.5	−2.50	2.5	−2.5	−2.5	−2.5
*R* ^2^	0.934	0.910	0.75	0.82	0.91	0.73	0.89
Normalized equation	$S=\frac{1}{\left(1+{\left(x/0.20\right)}^{2.5}\right)}$	$S=\frac{1}{{1+\left(x/0.20\right)}^{-2.50}}$	$S=\frac{1}{{1+\left(x/53.99\right)}^{-2.50}}$	$S=\frac{1}{1+{\left(x/0.015\right)}^{2.5}}$	$S=\frac{1}{{1+\left(x/4.91\right)}^{-2.5}}$	$S=\frac{1}{{1+\left(x/1.67\right)}^{-2.5}}$	$S=\frac{1}{{1+\left(x/34.66\right)}^{-2.5}}$
