# Supplementary material for: Restoration of Degraded Soil in the Nanmangalam Reserve Forest with Native Tree Species: Effect of Indigenous Plant Growth-Promoting Bacteria
Source: ScientificWorldJournal. 2016 Apr 18;2016:5465841. doi: 10.1155/2016/5465841 (PMC4852349; doi:10.1155/2016/5465841)
Supplement: Supplementary file 1 — Supplementary Table 1: Plants' Scientific name and their common name in Nanmangalam Reserve Forest. Supplementary Table 2: Correlation matrix between the different properties determined. Supplementary Figure 1: pH, EC, SOC, TN, MBC/MBN, and soil respiration values obtained for rhizosphere soil samples of the 12 different tree species. Supplementary Figure 2: Rhizosphere soil enzymes (urease, phasphatase, β-Glucosidase, dehydrogenase, phenoloxidase, Catalase) levels under the 12 different tree species. [file 5465841.f1.zip › S Table 1.docx]

**S. Table 1 Plants Scientific name and their common name in Nanmangalam Reserve Forest**

| **S.No** | **Plant Scientific Name** | **Common Name** |
| --- | --- | --- |
| 1 | *Albizia lebbeck* | Indian siris |
| 2 | *Azadirachta indica* | Neem tree, Indian lilac, Margosa tree |
| 3 | *Gmelina arborea* | gamhar, gumhar, gamari, beechwood, goomar teak, Kashmir tree, Malay beechwood, white teak |
| 4 | *Madhuca latifolia* | moha,mahua,madurgam |
| 5 | *Pongamia pinnata* | Indian Beech, Poongam Oil Tree, Honge, Ponge |
| 6 | *Pterocarpus santalinus* | Red Sandal |
| 7 | *Syzygium cumini* | Java plum, Malabar plum and Portuguese plum |
| 8 | *Tamarindus indica* | Tamarind |
| 9 | *Terminalia arjuna* | Arjuna, Koha, Kahu |
| 10 | *Terminalia bellirica* | Baheda, Belliric Myrobalan, Bastard myrobalan, Beach almond |
| 11 | *Thespesia populnea* | Portia tree, Indian tulip tree, Aden apple |
| 12 | *Wrightia tinctoria* | Sweet Indrajao, Pala indigo |
