# Supplementary material for: Restoration of Degraded Soil in the Nanmangalam Reserve Forest with Native Tree Species: Effect of Indigenous Plant Growth-Promoting Bacteria
Source: ScientificWorldJournal. 2016 Apr 18;2016:5465841. doi: 10.1155/2016/5465841 (PMC4852349; doi:10.1155/2016/5465841)
Supplement: Supplementary file 1 — Supplementary Table 1: Plants' Scientific name and their common name in Nanmangalam Reserve Forest. Supplementary Table 2: Correlation matrix between the different properties determined. Supplementary Figure 1: pH, EC, SOC, TN, MBC/MBN, and soil respiration values obtained for rhizosphere soil samples of the 12 different tree species. Supplementary Figure 2: Rhizosphere soil enzymes (urease, phasphatase, β-Glucosidase, dehydrogenase, phenoloxidase, Catalase) levels under the 12 different tree species. [file 5465841.f1.zip › S Table 2.docx]

**S. Table 2 Correlation matrix between the different properties determined**

| Properties | EC | SOC | TN | SBR | MBC | MBN | MBC/MBN | MBC/SOC | SBR/MBC | Urease | Phas | β- glucosidase | PO | Dehydrogenase | Catalase |
| --- | --- | --- | --- | --- | --- | --- | --- | --- | --- | --- | --- | --- | --- | --- | --- |
| EC | 1.00 | 0.58** | 0.27 | 0.36* | 0.12 | 0.26 | 0.19 | -.0.07 | 0.29 | 0.31 | 0.57** | 0.29 | 0.02 | 0.56** | 0.49** |
| SOC |  | 1.00 | 0.65** | 0.54** | 0.31 | 0.45* | -0.30* | -0.14 | 0.16 | 0.42* | 0.32 | 0.45** | 0.34* | 0.88** | 0.39* |
| TN |  |  | 1.00 | 0.29 | 0.34* | 0.56** | -.0.31 | -0.31 | 0.19 | 0.85** | 0.60** | 0.57** | 0.74** | 0.37* | 0.80** |
| SBR |  |  |  | 1.00 | 0.23 | 0.56** | -0.09 | -0.31 | 0.29 | 0.39* | 0.28 | 0.36* | 0.41* | 0.57** | 0.41** |
| MBC |  |  |  |  | 1.00 | 0.45* | 0.18 | 0.36* | 0.12 | 0.45 | 0.56** | 0.89** | 0.31 | 0.45* | 0.34* |
| MBN |  |  |  |  |  | 1.00 | 0.23 | -0.12 | 0.23 | 0.34 | 0.45* | 0.25 | 0.56** | 0.29 | 0.67** |
| MBC/MBN |  |  |  |  |  |  | 1.00 | -0.21 | 0.23 | -0.31 | -0.41* | 0.23 | 0.12 | 0.34* | -0.09 |
| MBC/SOC |  |  |  |  |  |  |  | 1.00 | 0.46* | 0.23 | 0.87** | 0.93** | 0.45* | 0.67** | 0.24 |
| SBR/MBC |  |  |  |  |  |  |  |  | 1.00 | 0.09 | 0.51** | 0.45* | 0.19 | 0.34* | 0.19 |
| Urease |  |  |  |  |  |  |  |  |  | 1.00 | 0.29 | 0.51** | 0.67** | 0.18 | 0.12 |
| Phosphatase |  |  |  |  |  |  |  |  |  |  | 1.00 | 0.45* | 0.34* | 0.29 | 0.31 |
| β- glucosidase |  |  |  |  |  |  |  |  |  |  |  | 1.00 | 0.12 | 0.35* | 0.91** |
| Phenol oxidase |  |  |  |  |  |  |  |  |  |  |  |  | 1.00 | 0.89** | 0.87** |
| Dehydrogenase |  |  |  |  |  |  |  |  |  |  |  |  |  | 1.00 | 0.34* |
| Catalase |  |  |  |  |  |  |  |  |  |  |  |  |  |  | 1.00 |

EC- Electrical conductivity, SOC- Soil organic carbon, TN- Total nitrogen, SBR- Soil basal respiration, MBC- Microbial biomass carbon, MBN- Microbial biomass nitrogen, Phas- Phosphatase, PO- Phenol-oxidase

*Correlation is significant at the p≤0.05 level (2- tailed); ** Correlation is significant at the p≤0.01 level (2- tailed)
