# Supplementary material for: Restoration of Degraded Soil in the Nanmangalam Reserve Forest with Native Tree Species: Effect of Indigenous Plant Growth-Promoting Bacteria
Source: ScientificWorldJournal. 2016 Apr 18;2016:5465841. doi: 10.1155/2016/5465841 (PMC4852349; doi:10.1155/2016/5465841)
Supplement: Supplementary file 1 — Supplementary Table 1: Plants' Scientific name and their common name in Nanmangalam Reserve Forest. Supplementary Table 2: Correlation matrix between the different properties determined. Supplementary Figure 1: pH, EC, SOC, TN, MBC/MBN, and soil respiration values obtained for rhizosphere soil samples of the 12 different tree species. Supplementary Figure 2: Rhizosphere soil enzymes (urease, phasphatase, β-Glucosidase, dehydrogenase, phenoloxidase, Catalase) levels under the 12 different tree species. [file 5465841.f1.zip › S_Figure_1_TSWJ_1640804.docx]

**S. Figure 1. pH, EC, SOC, TN, MBC/MBN, and soil respiration values obtained for rhizosphere soil samples of the 12 different tree species.**
